# Comparative plastomics of Amaryllidaceae: inverted repeat expansion and the degradation of the *ndh* genes in *Strumaria truncata* Jacq.

**DOI:** 10.7717/peerj.12400

**Published:** 2021-11-12

**Authors:** Kálmán Könyves, Jordan Bilsborrow, Maria D. Christodoulou, Alastair Culham, John David

**Affiliations:** 1 Royal Horticultural Society Garden Wisley, Woking, United Kingdom; 2 Herbarium, School of Biological Sciences, University of Reading, Reading, United Kingdom; 3 Department of Statistics, University of Oxford, Oxford, United Kingdom

**Keywords:** Amaryllidaceae, IR expansion, *ndh* loss, *Strumaria*

## Abstract

Amaryllidaceae is a widespread and distinctive plant family contributing both food and ornamental plants. Here we present an initial survey of plastomes across the family and report on both structural rearrangements and gene losses. Most plastomes in the family are of similar gene arrangement and content however some taxa have shown gains in plastome length while in several taxa there is evidence of gene loss. *Strumaria truncata* shows a substantial loss of *ndh* family genes while three other taxa show loss of *cemA*, which has been reported only rarely. Our sparse sampling of the family has detected sufficient variation to suggest further sampling across the family could be a rich source of new information on plastome variation and evolution.

## Introduction

The plastid genome, or plastome, in land plants is generally conserved in length, structure, and gene content (Wicke et al., 2011). Typical flowering plant plastomes range from 120 to 160 kb, contain 100–120 unique genes, and have a quadripartite structure of two single copy regions (LSC and SSC) separated by two copies of the inverted repeat (IRs) (Jansen & Ruhlman, 2012; Smith & Keeling, 2015). However, exceptions to all of these features have been found. The greatly reduced plastomes of *Pilostyles* range from 11–15 kb and contain only seven functioning genes (Arias-Agudelo et al., 2019). In contrast the plastome of *Pelargonium* × *hortorum* has expanded to 218 kb including unusually long inverted repeats (Chumley et al., 2006). Plastomes deviating from the quadripartite structure have also been reported, either without one copy of the IR (Wojciechowski et al., 2000; Sanderson et al., 2015) or incorporating the entire SSC into the inverted repeats (Sinn et al., 2018).

Rearrangements are often associated with increased repeat content in plastid genomes (Chumley et al., 2006; Haberle et al., 2008; Guisinger et al., 2011). One of the most commonly reported plastome rearrangements is the expansion or contraction of the inverted repeats. Palmer et al. (1985) and Yamada (1991) hypothesised that intramolecular recombination at short inverted repeats located within and around the main IR resulted in the expansion of the *Chlamydomonas reinhardtii* and *Chlorella ellipsoidea* inverted repeats, respectively, while Aii et al. (1997) proposed that recombination at forward repeats within *ycf1* lead to an inversion, and the IR expansion in buckwheat (*Fagopyrum* sp.). However, in the absence of short repeats in *Nicotiana*, Goulding et al. (1996) established two different mechanisms for both short, <100 bp, and long, over 10 kb, IR expansions: short expansions are the result of gene conversion after heteroduplex formation *via* Holliday junctions, and long expansions are the result of a double-strand break (DSB) in one of the IRs followed by strand invasion and repair against the other IR within the same plastome unit that progresses through the junction incorporating single-copy regions into the inverted repeats. A similar DSB repair process, but *via* homologous recombination at imperfect nonallelic repeats between different plastome units, resulted in the reestablishment of the IR in *Medicago* (Choi, Jansen & Ruhlman, 2019). Zhu et al. (2016) attributed the IR expansions in several dicot lineages to the gene conversion mechanism. Furthermore, Wang et al. (2008) proposed that the DSB mechanism of Goulding et al. (1996) can account for shorter expansions as well, such as the development of the monocot-type IR/LSC junction by incorporating *rps19* and *trnH* into the IR.

Various gene losses have occurred during the evolution of the angiosperm plastome (Raubeson & Jansen, 2005; Jansen et al., 2007); a commonly reported example is the loss of *ndh* genes (Song et al., 2017; Silva et al., 2018; Nevill et al., 2019). The loss of *ndh* genes is strongly associated with a change in trophic conditions (Wicke et al., 2013; Graham, Lam & Merckx, 2017); it represents the first step in plastome degradation in heterotrophic plants (Martín & Sabater, 2010; Barrett & Davis, 2012). Apart from plant lineages less reliant on photosynthesis, multiple *ndh* gene losses have also been reported in fully photosynthetic lineages for example in aquatic/semi-aquatic plants (Iles, Smith & Graham, 2013; Peredo, King & Les, 2013; Folk et al., 2020), gymnosperms (Wakasugi et al., 1994; McCoy et al., 2008), Orchidaceae (Kim et al., 2015; Roma et al., 2018), and Cactaceae (Sanderson et al., 2015; Köhler et al., 2020).

Amaryllidaceae J. St.-Hil. (Saint-Hilaire, 1805) is a cosmopolitan family of bulbous geophytes and rhizomatous perennials in Asparagales (Meerow, 2000) comprising approximately 90 genera, and over 1,700 species (Meerow, Gardner & Nakamura, 2020), in three subfamilies: Amaryllidoideae, Allioideae, and Agapanthoideae, all of which share an umbellate inflorescence. This family of petaloid monocots contains many horticulturally important genera, including: *Agapanthus, Allium, Amaryllis, Clivia, Galanthus, Hippeastrum, Narcissus,* and *Nerine* (Heywood et al., 2007). Amaryllidoideae, the most diverse subfamily with c. 75 genera (Meerow, Gardner & Nakamura, 2020), has a complex evolutionary history, including hybridisation (García et al., 2017; Marques et al., 2017; Meerow, Gardner & Nakamura, 2020), and morphological convergence (Meerow, 2010). Arising from our research, in horticulturally important Amaryllidaceae genera (Könyves et al., 2018; Könyves et al., 2019a; David & Könyves, 2019), here we report the sequencing and assembly of five Amaryllidoideae species and compare our assemblies with available Amaryllidaceae plastomes from GenBank. This will broaden the knowledge, and reporting, of plastome structural variation in Amaryllidaceae.

## Materials & Methods

Fresh leaf material was collected from five Amaryllidoideae species at RHS Garden Wisley, UK or from a private collection (Table 1). Our sampling was fundamentally opportunistic, including Amaryllidoideae species that were growing at the time of collection, and related to other projects we were working on (Könyves et al., 2018; Könyves et al., 2019a; David & Könyves, 2019). It did, however, broaden the generic level sampling of the subfamily because these genera were chosen to act as outgroups to other, generic-level studies. Herbarium voucher specimens were deposited at WSY. Total genomic DNA was extracted using the QIAGEN DNeasy Plant Mini Kit (QIAGEN, Manchester, UK). Library development and 150 bp PE (paired-end) sequencing on an Illumina HiSeq 4000 lane was done by the Oxford Genomics Centre (Oxford, UK). The plastomes were assembled with Fast-Plast v1.2.6 (McKain & Wilson, 2017) and NOVOPlasty v2.7.0 (Dierckxsens, Mardulyn & Smits, 2017). Fast-Plast assemblies were run with a total of 5M, 10M, 20M reads (*i.e.,* 2.5M, 5M, 10M PE reads) and with all available reads. Reads were trimmed to remove NEB-PE adapter sequences. Bowtie reference indices were built with the published *Narcissus poeticus* plastome (MH706763). For the NOVOPlasty assemblies, adapters were trimmed with Trimmomatic v0.36 (Bolger, Lohse & Usadel, 2014), using the same adapter sequences. An *ndhF* sequence of *Na. poeticus* (KT124416) was used as the starting seed and memory was limited to 8 Gb. All other parameters were unchanged. The *Strumaria truncata* NOVOPlasty assembly failed with the *ndhF* seed, a *trnK/matK* sequence of *Na. poeticus* (KC238498) was used instead. Among those assemblies that did not produce consistent results across the different assembly strategies, the large single copy (LSC), the small single copy (SSC), and two inverted repeat (IR) regions were identified in the final Fast-Plast contig and NOVOPlasty assemblies, and the circular plastome was assembled by hand using Geneious v11.1.5 (http://www.geneious.com; Kearse et al., 2012). The junctions of the inverted repeats and the *ndh* gene sequences in the *S. truncata* plastome assembly were confirmed by Sanger sequencing using the primers and PCR protocols detailed in Tables S1 and S2. Coverage analysis of the finished plastomes were done in Fast-Plast. The complete plastomes were annotated by transferring equivalent annotations from the *Na. poeticus* plastome using Geneious v11.1.5. Gene and exon boundaries were corrected by hand when necessary.

**Table 1:** Source information and GenBank accession numbers of the samples included in this study. Herbarium voucher codes (WSY) and publications are listed when available.

**Species**	**GenBank acc. no.**	**Source**
*Acis autumnalis* var. *oporantha* (Jord. & Fourr.) Lledó, A.P.Davis & M.B.Crespo	MN539611	This study (WSY0153095)
*Lapiedra martinezii* Lag.	MN539612	This study (WSY0153095)
*Nerine sarniensis* (L.) Herb.	MN539613	This study (WSY0153096)
*Pancratium maritimum* L.	MN539614	This study (WSY0153097)
*Strumaria truncata* Jacq.	MN539615	This study (WSY0153098)
*Clivia miniata* (Lindl.) Verschaff.	MN857162	GenBank (Wang et al., 2020)
*Hippeastrum rutilum* (Ker Gawl.) Herb.	MT133568	GenBank (Huang, 2020)
*Leucojum aestivum* L.	MH422130	GenBank (Li et al., 2018)
*Lycoris radiata* (L’Hér.) Herb.	MN158120	GenBank (Zhang et al., 2019)
*Lycoris squamigera* Maxim.	MH118290	GenBank (Jin et al., 2018)
*Narcissus poeticus* L.	MH706763	GenBank (Könyves et al., 2018)
*Allium altaicum* Pall.	MH159130	GenBank ( Filyushin et al., 2018)
*Allium fistulosum* L.	MH926357	GenBank (Yusupov et al., 2019)
*Allium praemixtum* Vved.	MK411817	GenBank (Yusupov et al., 2021)
*Agapanthus coddii* F.M.Leight.	KX790363	GenBank (unpublished)
*Hesperoyucca whipplei* (Torr.) Trel.	KX931459	GenBank (McKain et al., 2016)
*Hemerocallis fulva* (L.) L.	MG914655	GenBank (Lee et al., 2019)

The plastomes constructed in this study were combined with ten plastomes representing all three subfamilies of Amaryllidaceae, and a further two Asparagales (Asparagaceae and Asphodelaceae) plastomes from GenBank (Table 1). Sixty-seven coding genes (CDS) that are shared between all samples were extracted from the whole plastomes and aligned with the MUSCLE algorithm v3.8.425 using the default parameters in Geneious Prime 2020.0.5 (http://www.geneious.com). Prior to alignment, annotations of GenBank sequences were amended to correct reading frames, where necessary (changes listed in Table S3). The alignments of the 67 CDS were concatenated into a matrix of 58,230 bp. A maximum likelihood estimate of phylogeny was conducted with RAxML v8.2.11 (Stamatakis, 2014) within Geneious Prime using 1000 bootstrap replicates according to the best-fit model of evolution, GTR+I+G, identified by jModelTest 2 (Guindon & Gascuel, 2003; Darriba et al., 2012).

**Figure 1:**
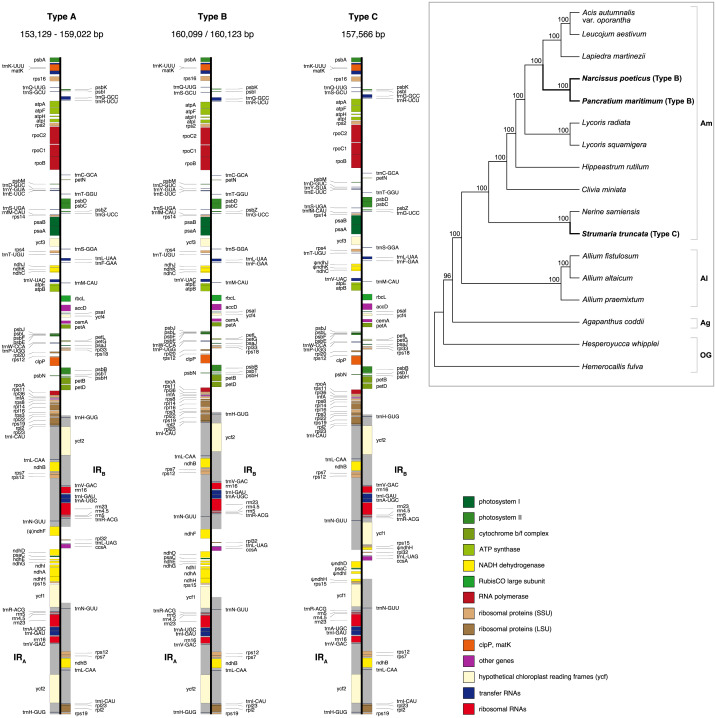
Maps of the three types of plastomes characterised by the 5′ portion of *ycf1* present in IR_A_. Gray shading highlights the IR regions. Genes are coloured according to functional groups shown in the legend. Inset panel shows the relationship between the sampled species based on the RAxML analysis of 67 coding sequences. Bootstrap support values are shown at nodes. Type B and C plastomes are highlighted in bold, all other samples had Type A plastomes. Amaryllidaceae subfamilies are indicated on the right: Am, Amaryllidoideae; Al, Alliodideae; Ag, Agapanthodieae; OG, Outgroups.

**Figure 2:**
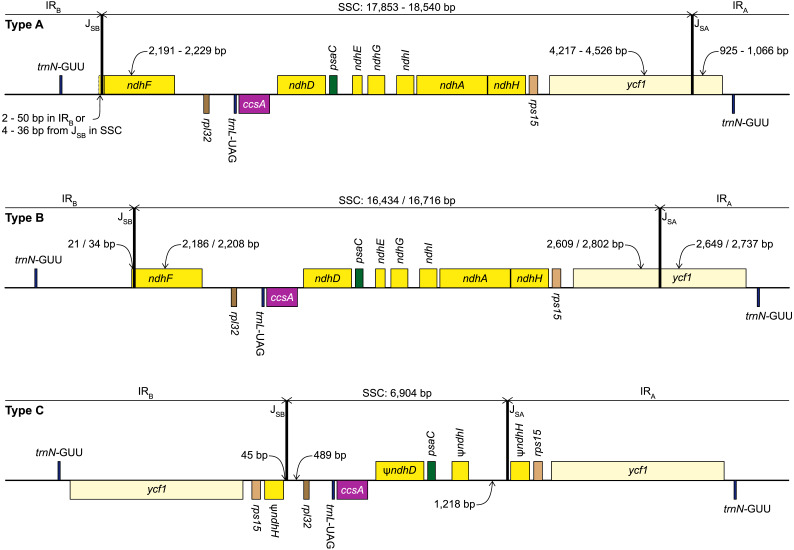
Structure of the junctions (J_SA_, J_SB_) between the inverted repeats (IR_A_, IR_B_) and small single copy (SSC) in the three plastome types identified in this study. The portion of genes included in the IRs and SSC is indicated. Gene order, direction of transcription, and colour code of each gene correspond to Fig. 1. *ψ* indicates pseudogenes.

The inverted repeat boundaries for all samples were identified using the Repeat Finder v1.0.1 plugin in Geneious Prime, with default settings. We classified the plastomes into three groups (Type A, B, C) based on the 5′ portion of *ycf1* present in IR_A_, *i.e.,* the structure of the IR_A_-SSC junction (J_SA_), to identify IR expansion events. We did not identify any inversions in the plastomes therefore we tested whether the IR_A_-SSC expansions could have happened through recombination at short inverted repeats present, *i.e.,* the mechanism proposed by Palmer et al. (1985) and Yamada (1991), or through short forward repeats which could mediate recombination as mentioned by Choi, Jansen & Ruhlman (2019). We searched for short repeats present in both target regions in each species of interest, detailed below, using the Repeat Finder v1.0.1 plugin in Geneious Prime with a minimum of 16 bp length and allowing 10% mismatch. We chose the minimum length and allowed for mismatches as Staub & Maliga (1994) showed evidence of recombination at such repeats in plastids. In *Na. poeticus* and *Pancratium maritimum* (plastome Type B) we screened the region 500–1,500 bp from the 5′ end of *ycf1* (the position where J_SA_ in Type A plastomes occurs, see Figs. 1 and 2) and the 500 bp either side of J_SA_ in both species. Repeat Finder did not identify any repeats in *Na. poeticus* so we manually checked the target regions for those homologous with *P. maritimum* to see if this lack is due to our strict search settings. To further investigate the loss of the *ndh* genes in *S. truncata*, we screened for the repeats in *Nerine sarniensis* as well, 500 bp either side of J_SA_ and within *ndhH*, where J_SA_ in *S. truncata* is found. The inverted repeat in *S. truncata* contains a 45 bp region downstream of the *ndhH* pseudogene that is absent in other taxa. To identify where this 45 bp region originated we aligned a portion of the *S. truncata* plastome, spanning from *trnN* in IR_B_ to *trnN* in IR_A_, against the same portion of the *Ne. sarniensis* assembly using the MUSCLE algorithm with the default parameters in Geneious Prime. We identified tandem repeats using Phobos v3.3.12 (Mayer, 2006–2010), within Geneious Prime, following Joyce et al. (2019), by restricting the search to perfect repeats between 2 and 1,000 bp long, with the “remove hidden repeats” setting enabled.

Protein coding genes were categorised as intact, putatively pseudogenised, or lost following Joyce et al. (2019). Briefly, genes were annotated as putative pseudogenes if they contained internal stop codons, or the stop codon was missing. Genes were considered lost if less than 30% of the gene was present compared with the other samples. Plastome maps were drawn in OGDRAW v1.3.1 (Greiner et al., 2011).

## Results

Illumina pair-end sequencing for the samples in this study produced 19,015,437 - 24,547,050 raw paired-end reads. All assembly strategies produced consistent assemblies for *P. maritimum*. Fast-Plast with 20M reads and NOVOPlasty assemblies for *Acis autumnalis* var. *oporantha* were congruent. Variation in strategies for assembly for all other plastomes caused differences in overall length (Table S4), likely due to differing assembly of repeat rich regions. Notwithstanding, the structural parts of the plastome (LSC-IR-SSC) and expected genes could be identified in all output files, therefore final assemblies were constructed by hand. Sanger sequencing confirmed that the junctions in all five plastomes and the *ndh* genes in *S. truncata* were correctly assembled. Average coverage of the final assemblies ranged from 786 × to 1, 379 × (Table S4). Raw sequence data are available in SRA (BioProject: PRJNA730513); assembled plastomes are available on GenBank (MN539611–MN539615). Amaryllidaceae plastomes (Fig. 1) have a quadripartite structure, range from 153,129 to 160,123 bp in length, and contain 70–86 protein coding genes, 38 tRNAs and 8 rRNAs (Table 2).

**Table 2:** Comparison of plastome features in Amaryllidaceae and outgroup samples. Plastomes assembled in this study are in bold.

	Amaryllidaceae															Asparagaceae	Asphodelaceae
	Amaryllidoideae											Allioideae			Agapanthoideae		
	***Acis autumnalis*** **var.** ***oporantha***	***Lapiedra martinezii***	***Nerine sarniensis***	***Pancratium maritimum***	***Strumaria truncata***	*Clivia miniata*	*Hippeastrum rutilum*	*Leucojum aestivum*	*Lycoris radiata*	*Lycoris squamigera*	*Narcissus poeticus*	*Allium altaicum*	*Allium fistulosum*	*Allium praemixtum*	*Agapanthus coddii*	*Hesperoyucca whipplei*	*Hemerocallis fulva*
GenBank accession number	**MN539611**	**MN539612**	**MN539613**	**MN539614**	**MN539615**	MN857162	MT133568	MH422130	MN158120	MH118290	MH706763	MH159130	MH926357	MK411817	KX790363	KX931459	MG914655
Total plastome length (bp)	157,839	159,022	158,312	160,123	157,566	158,114	158,357	157,241	158,335	158,459	166,099	153,129	153,164	153,226	157,055	157,832	155,855
LSC length (bp)	85,792	86,756	86,271	86,393	85,648	86,204	86,451	85,657	86,613	86,431	86,445	82,196	82,237	82,162	85,204	86,170	84,607
SSC length (bp)	18,367	18,540	18,455	16,716	6,904	18,334	18,272	18,180	18,262	18,500	16,434	17,913	17,907	18,042	18,113	18,228	18,508
IR length (bp)	26,840	26,863	26,793	28,507	32,507	26,788	26,817	26,702	26,730	26,764	28,610	26,510	26,510	26,511	26,869	26,717	26,370
Overall G/C content	37.7	37.8	37.8	37.8	37.8	38	37.9	37.9	37.8	37.8	37.8	36.8	36.8	36.8	37.5	37.8	37.4
Number of tandem repeats	562	605	601	556	591	568	560	567	579	572	543	553	564	561	570	576	666
Sum length of tandem repeats (bp)	7,017	7,939	7,922	6,875	7,591	7,024	7,021	7,178	7,140	7,029	6,838	7,066	7,267	7,254	6,975	7,019	8,790
Percentage length of tandem repeats	4.45	4.99	5.00	4.29	4.82	4.44	4.43	4.56	4.51	4.44	4.12	4.61	4.74	4.73	4.44	4.45	5.64
N of Intact protein coding genes (unique)	86 (79)	86 (79)	84 (77)	85 (78)	79 (70)	86 (79)	86 (79)	86 (79)	85^*^ (79)	86 (79)	85 (78)	85 (78)	85 (78)	85 (78)	86 (79)	86 (79)	86 (79)
Pseudo protein coding genes	0	0	2	1	5	0	0	0	0	0	1	1	1	1	0	0	0
Lost protein coding genes	0	0	0	0	4	0	0	0	0	0	0	0	0	0	0	0	0
N of Intact tRNA (unique)	38 (30)	38 (30)	38 (30)	38 (30)	38 (30)	38 (30)	38 (30)	38 (30)	38 (30)	38 (30)	38 (30)	38 (30)	38 (30)	38 (30)	38 (30)	38 (30)	38 (30)
N of Intact rRNA (unique)	8 (4)	8 (4)	8 (4)	8 (4)	8 (4)	8 (4)	8 (4)	8 (4)	8 (4)	8 (4)	8 (4)	8 (4)	8 (4)	8 (4)	8 (4)	8 (4)	8 (4)

**Notes.**

* *rps19* in IR_A_ of *Lycoris radiata* is truncated.

We identified three different J_SA_ structures (Fig. 1) in our samples. Type A was the most frequent junction type, found in 14 samples, and most plausibly the ancestral IR junction in Amaryllidaceae, as it is shared with both outgroups. The other two J_SA_ structures show two independent IR expansion events. Type B, present in *Na. poeticus* and *P. maritimum*, is a shared IR expansion event, while Type C in *S. truncata* represents a different IR expansion. The 5′ portion of *ycf1* within the IR_A_ ranged from 925–1,066 bp for Type A (Fig. 2). The Type B plastome had 2,649 or 2,737 bp of *ycf1* included in IR_A_. The IR expansion in Type C has a different gene arrangement (Fig. 2): the entire *ycf1*, *rps15*, and pseudogenised *ndhH* were included in the IR. The IRs and the SSC ranged from 17,853 to 18,540 bp and 26,370 to 26,869 bp, respectively, in Type A plastomes. The IRs in the Type B plastomes were longer, at 28,507 or 28,610 bp, with a shorter SSC, of 16,434 or 16,716 bp. The expanded IRs in *S. truncata* (Type C) were the longest at 32,507 bp, while the 6,904 bp long SSC was the shortest. The arrangement of the junction between SSC and IR_B_ also showed variation in Type A and Type B plastomes, however this was due to the length variation of the 3′ end of *ndhF* rather than IR expansion/contraction.

**Figure 3:**
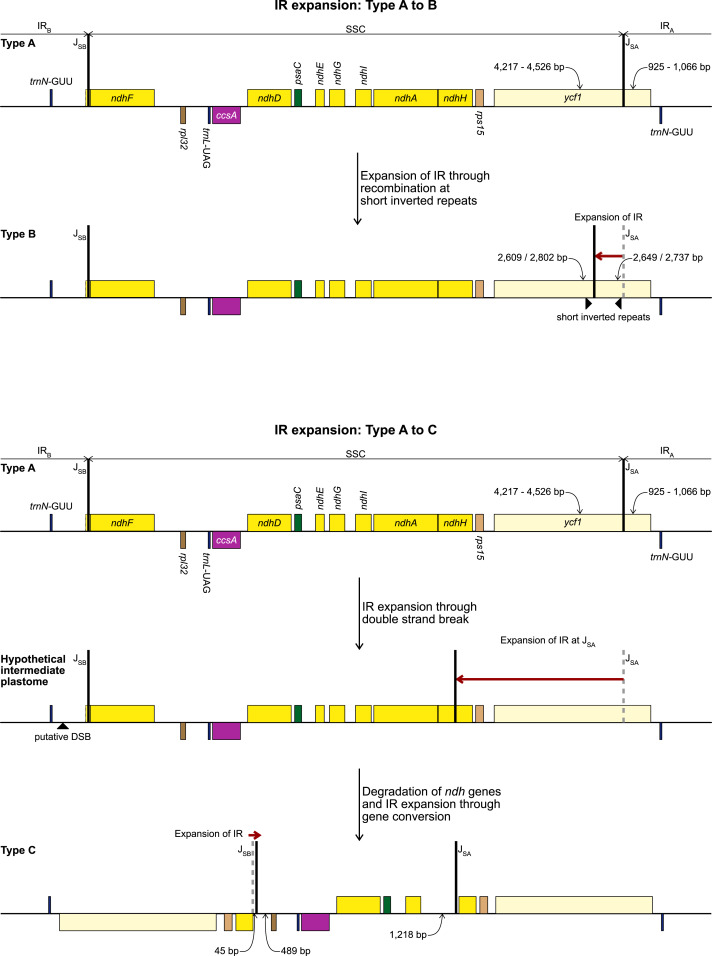
Development of Type B (top) and Type C (bottom) plastome from the ancestral Type A plastome. Steps required to explain the structural changes and the putative mechanisms are detailed in the figure. Gene order, direction of transcription, and colour code of each gene correspond to Fig. 1.

Repeat Finder identified only one short inverted repeat, with our search parameters, in *P. maritimum* (Table 3). A similar repeat is present in the *Na. poeticus* plastome, however this has more mismatches than the 10% threshold we set for the search. Both Type B plastomes contain the same, short, forward repeat, but in *Na. poeticus* this repeat is only partially in our target region. No short repeats were identified in the *S. truncata* or *Ne. sarniensis* plastomes. Figure 3 summarises the putative mechanisms for the development of the Type B and C plastomes.

**Table 3:** Details of short repeats present at J_SA_ in *Pancratium maritimum* and *Narcissus poeticus*.

**Species**	**Repeat sequence**	**Direction**	**Repeat length**	**Position**	**Mismatched bases**
*P. maritimum*	TCATTATTAGGTTTATA - TATAAACCCAATAATGA	inverted	17 bp	131,409–131,425 133,062–133,078	1
*P. maritimum*	TTCATTTTTCTCTTCTTT - TTCATTTTCCTCTTCTTT	forward	18 bp	132,022–132,039 133,345–13,336	1
*Na. poeticus*	TCATTATTAGGTTTATA - TATAAACCCAATAATTA	inverted	17 bp	131,379–131,395 133,023–133,039	2
*Na. poeticus*	TTCATTTTTCTCTTCTTT - TTCATTTTCCTCTTCTTT	forward	18 bp	131,983–132,000 133,306–133,323	1

The alignment of the regions between the two copies of *trnN* encompassing the SSC from *Ne. sarniensis* and *S. truncata* showed that the 45 bp present in the *S. truncata* IR and the following single-copy sequence towards *rpl32* is homologous with the *ndhF-rpl32* spacer in *Ne. sarniensis* (Fig. S1). These 45 bp represent a second short independent IR expansion in *S. truncata* (Fig. 3).

**Figure 4:**
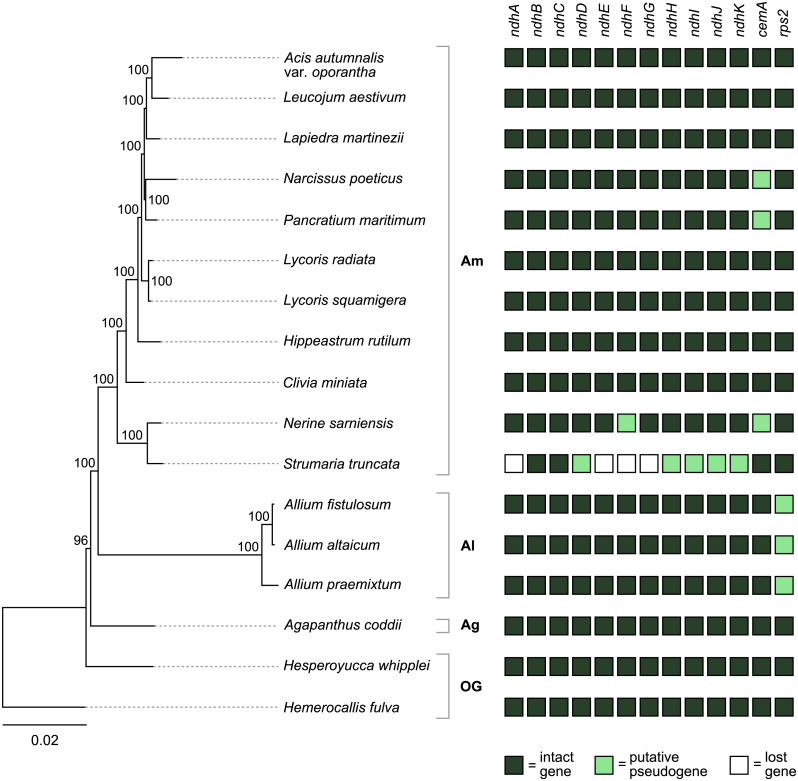
Pattern of gene losses and pseudogenization in Amaryllidaceae and the two outgroup samples plotted against the RAxML tree based on 67 coding sequences. Bootstrap support values are shown at nodes. Plastomes assembled in this study are highlighted in bold. Amaryllidaceae subfamilies are indicated on the right: Am, Amaryllidoideae; Al, Alliodideae; Ag, Agapanthodieae; OG, Outgroups.

The *S. truncata* plastome (Type C) showed a degradation of the *ndh*-suite (Fig. 4). Only two *ndh*-genes remained intact, *ndhB* and *ndhC*. The *ndhA*, *ndhE*, *ndhF*, and *ndhG* genes were lost, while *ndhD*, *ndhH*, *ndhI*, *ndhJ*, *ndhK* were pseudogenised. The combined length of the two putative exons of *ndhA* is 21% of the length of *ndhA* in other Amaryllidaceae samples. The sequence regions of the other genes classified as lost (*ndhE, ndhF, ndhG*) were entirely absent. The *ndhH* gene is 1,182 bp long in Amaryllidaceae. In *S. truncata*, this gene is included in the IR as two putative pseudogenes, each copy is only 592 bp long (50%) from the start codon, and the stop codons are missing. The *ndhJ* gene contained an internal stop codon due to transversion mutation (G to T at 49,624 bp), all other putative pseudogenes had frameshift mutations. Furthermore, *ndhF* was classified as a putative pseudogene in *Ne. sarniensis* due to a missing stop codon. The *ndhC* gene in *Leucojum aestivum* is missing 47 bp, including the start codon, at the 5′ end of the gene preceded by 2 N’s, potentially indicating scaffolded contigs due to missing data. We did not categorise this gene as a putative pseudogene due to this potential missing data, however we did exclude the gene from the phylogenetic analysis. The *cemA* gene contains frameshift mutations in *Na. poeticus*, *Ne. sarniensis*, and *P. maritium* due to a homopolymeric A repeat at the 5′ end of the gene. In all *Allium* samples *rps2* is a pseudogene.

The number of tandem repeats and their length as percentage of the total plastome ranged from 543 to 605 and 4.1% to 5.6%, respectively, in Amaryllidaceae (Table 2). Tandem repeat numbers in Type A plastomes ranged from 560 to 601 (4.4–5.0%) (Table 2). Type B plastomes had 543 (4.1%), and 556 (4.3%) tandem repeats in *Na. poeticus* and *P. maritimum*, respectively, and 591 (4.8%) in *S. truncata* (Type C). Moreover, the highest number of repeats, 666, was found in *Hemerocallis fulva*, which shows no IR expansion. These changes indicate that an increased number of tandem repeats did not contribute to the IR expansions in Type B and C plastomes.

The phylogenetic analysis based on 67 protein coding genes recovered a fully resolved phylogenetic tree with maximum (100%) bootstrap support for all branches except for the one leading to the ingroup (96%, Fig. 4). Within Amaryllidaceae, Agapanthoideae is sister to Allioideae and Amaryllidoideae. Within Amaryllidoideae, *Ne. sarniensis* is sister to *S. truncata* and in turn they are sister to the remaining Amaryllidoideae species. Sequence alignment of the 67 protein coding genes used in the phylogenetic analysis and the resulting newick tree is available at: https://github.com/kalmankonyves/Amaryllidaceae_plastomes-Strumaria.

## Discussion

The newly sequenced and assembled plastomes of five genera from Amaryllidoideae exhibited three plastome arrangement types based on the portion of *ycf1* within the inverted repeats. Previously published plastomes such as *Allium* (Huo et al., 2019) and *Narcissus* (Könyves et al., 2018) fall into our Type A and B respectively, however the Type C plastome, with the largest IR expansion, represents a novel rearrangement in Amaryllidaceae that has not been reported previously.

In typical angiosperm plastomes ∼1,000 bp of *ycf1* is included in the IR (Sun et al., 2017). This is similar to the Type A plastome we recognised in Amaryllidaceae. We identify the Type A plastome as the ancestral state in the family, as it is shared with both outgroups (Fig. 1). The IR expansions that gave rise to the Type B and Type C plastomes represent independent events. In *Na. poeticus* and *P. maritimum* (Type B) the IR has expanded to include a larger portion of *ycf1* (2,649/2,737 bp), while in *S. truncata* (Type C) the whole of *ycf1* along with *rps15* and a pseudogenised *ndhH* are contained within the IR. The expansion or contraction of the inverted repeats have been shown to occur in multiple land plant lineages (Wicke et al., 2011; Jansen & Ruhlman, 2012; Zhu et al., 2016) and can often be specific to a few genera within a family (Guisinger et al., 2011; Dugas et al., 2015; Tian et al., 2018; Thode & Lohmann, 2019). In Asparagales IR expansions have been reported in multiple genera in Orchidaceae, incorporating genes from the SSC up to and including *ccsA* (Kim et al., 2015; Kim et al., 2020), and in *Eustrephus latifolius*, Asparagaceae (Kim, Kim & Kim, 2016), where the IR expanded to include *ycf1*.

Rearrangements in the plastomes have been associated with an increased number of repeats (Guisinger et al., 2011; Sinn et al., 2018). There was no correlation between the number of tandem repeats and IR expansion in Amaryllidaceae; the tandem repeat numbers and their length as a percentage of the total plastome (543–605; 4.1%–5.6%) are similar to those reported by Sinn et al. (2018) for non-rearranged plastomes (420–732; 3.3%–6.0%). However, we identified 17 bp inverted repeats in the vicinity of the IR junctions in both *P. maritimum* and *Na. poeticus*. We propose that the IR expansion in these species might have happened through recombination of similar short inverted repeats in the common ancestor of these genera (Fig. 3). The literature offers no consensus on how long short inverted repeats are. The earliest report by Palmer et al. (1985) for *Chlamydomonas reinhardtii*, identified 100–300 bp repeats in the vicinity of the IR junctions, however Aldrich et al. (1988) found 7 bp inverted repeats in *Petunia* and hypothesised that recombination at these repeats led to the IR expansion. We based our search for short inverted repeats on Staub & Maliga (1994) who showed experimental evidence of recombination at 16 bp imperfect repeats in *Nicotiana tabacum* resulting in extrachromosomal elements.

No short repeats were identified in the *S. truncata* or *Ne. sarniensis* plastomes. Therefore, we hypothesise that the IR expansion in *S. truncata* could be a result of a double-strand break repair (Fig. 3) as described by Goulding et al. (1996) in *Nicotiana* or homologous recombination between different plastome units could also have produced the expanded IR. Choi, Jansen & Ruhlman (2019) proposed that short non-allelic repeats mediated recombination in *Medicago* resulting in the reestablishment of the inverted repeats. As we did not find any suitable repeats, the recombination instead could have happened at homologous genes as shown by Ruhlman et al. (2017) in *Monsonia*. It is also possible that any site of recombination could have been lost during the degradation of the *ndh* genes. We identified a 45 bp region downstream of the annotated *ndhH* pseudogene to have originated in the *ndhF-rpl32* spacer. This indicates that the IR-SSC organisation of *S. truncata* is the result of two independent IR expansion events (Fig. 3). Most likely, first a double-strand break originating in IR_B_ got repaired against the complementary strand of IR_A_ with the copy-repair progressing beyond the IR_A_-SSC junction incorporating *ycf1*, *rps15*, and 592 bp of *ndhH* into the IR. A second small IR expansion originating in IR_B_ further expanded the IR incorporating 45 bp of the region upstream of *rpl32* through the gene conversion mechanisms of Goulding et al. (1996). Although Goulding et al. (1996) called the mechanism responsible for short IR expansion ‘gene conversion’, their description: “Branch migration of a Holliday junction across an IR/LSC junction results in the formation of heteroduplex before this process stalls. Resolution of heteroduplex may then proceed by sequence correction against either strand…” does not preclude this model from applying to expansions at SC/IR junctions with non-coding sequences. Furthermore, ‘gene conversion’ at non-coding sequences removes the constraint of maintaining functional genes.

In *S. truncata* nine out of the 11 *ndh* genes have been lost or pseudogenised (Fig. 4). Loss or pseudogenisation of *ndh* genes has been reported in Asparagales both in mycoheterotrophic and autotrophic orchids (Barrett et al., 2014; Kim et al., 2015; Kim et al., 2020; Roma et al., 2018), and in *Allium paradoxum* (2019). We found a further putative *ndh* pseudogene, *ndhF* in *Ne. sarniensis,* which is missing the stop codon. It is possible that this gene is functional and C-to-U RNA editing creates a stop codon at transcription. The pseudogenes in *S. truncata,* on the other hand, are the result of frameshift mutations which are not known to be edited in plastomes (Haberle et al., 2008). The *ndh* genes encode the NADH dehydrogenase-like complex (NDH-1) which regulate photosynthetic electron transport (Peltier, Aro & Shikanai, 2016; Shikanai, 2016). NDH-1 helps adapt photosynthesis under photooxidative stress conditions (Martín & Sabater, 2010) and in environments with fluctuating light intensity (Yamori & Shikanai, 2016). The loss of the *ndh* complex has been reported in plants growing either in high or low light level environments, for example hot desert (Sanderson et al., 2015), submersed (Peredo, King & Les, 2013), or understorey habitats (Graham, Lam & Merckx, 2017; Omelchenko et al., 2020). Furthermore, under optimal growth conditions *ndh* genes appear dispensable (Burrows et al., 1998; Endo et al., 1999; Rumeau, Peltier & Cournac, 2007), and their function could also be assumed by an alternate nuclear encoded system (Wicke et al., 2011) *e.g.*, the AA-sensitive CEF pathway (Sanderson et al., 2015). In the presence of an alternate system, the degradation of the *ndh* genes can have little consequence on plant fitness and mutations can freely accumulate leading to pseudogenisation and loss. Alternatively, *ndh* genes can be transferred to the nucleus and only lost from the plastome, as Roma et al. (2018) showed in *Ophrys.* The data we present in this paper, shallow sequencing of whole genomes (*e.g.*, ∼6.3 Gb of raw sequence data represent ∼0.3–0.5× coverage of the nuclear genome of *Ne. sarniensis:* 12.6/19.4 Gb; Leitch et al. (2019)), allows us to simply hypothesise the fate of pseudogenised and lost genes. The loss of *ndh* genes from the *S. truncata* plastome compared with *Ne. sarniensis* could be the result of ecological adaptation, as the two species occur in different habitats: *S. truncata* inhabits the semi-arid Succulent Karoo, while *Ne. sarniensis* occurs in the Fynbos, characterised by a wetter, Mediterranean-type climate (Duncan, Jeppe & Voigt, 2020). It is also possible that the loss of the *ndh* genes is a result of a transfer to the nucleus, or the presence of an alternate system. Further sampling of *Strumaria* and *Nerine* species and investigating the nuclear genome and transcriptome will be necessary to identify potential routes of gene loss from the plastome.

Kim et al. (2015) and Kim et al. (2020) showed a correlation between the presence of *ndhF* and the organisation of the IR-SSC junctions in Orchidaceae, and proposed that the loss of *ndhF* leads to destabilisation of the IR-SSC junctions. This holds true to *Najas flexilis*, mentioned by Kim et al. (2015), and could be supported by our results. However, there is also published evidence that IR expansion can happen without the loss of *ndh* genes: plastomes in Thymelaeaceae have all SSC genes, apart from *ndhF* and *rpl32,* incorporated in the IR in the presence of a full set of 11 *ndh* genes (Könyves et al., 2019b; Lee et al., 2020; Liang, Xie & Yan, 2020), and in *Asarum* the IR expanded to encompass the whole of the SSC without the loss of *ndh* genes (Sinn et al., 2018).

The IR expansion in *S. truncata* is plausibly the result of two independent events, therefore the order in which gene losses and expansions happened are difficult to unravel. It is perhaps more parsimonious to suggest that the degradation of the *ndh* genes or at least the loss of *ndhF* preceded the second IR expansion in *S. truncata*, otherwise the whole of *ndhF* had to be incorporated into the IR and subsequently lost from both IR_A_ and IR_B_. Further sampling in Amaryllidoideae could help establish the sequence of gene loss and IR expansion and any correlation between these events.

There were further pseudogenised genes found in Amaryllidaceae: the chloroplast envelope membrane protein encoding *cemA* is pseudogenised in *Ne. sarniensis*, *Na. poeticus*, and *P. maritimum* due to a homopolymeric A repeat adjacent to the potential initiation codon. A similar pattern of pseudogenisation has been shown in *Lilium* spp. (Do & Kim, 2019) and *Cocos nucifera* (Huang, Matzke & Matzke, 2013). The *cemA* gene is not essential for photosynthesis, however in high light conditions *cemA*-lacking mutants of *Chlamydomonas reinhardtii* showed decreased photosynthetic efficacy (Rolland et al., 1997). The *rps2* gene is functional in Amaryllidoideae and *Agapanthus coddii* but has pseudogenised due to internal stop codons in *Allium*, which has been reported previously by Filyushin, Beletsky & Kochieva (2019), Omelchenko et al. (2020) and Xie et al. (2019).

The phylogenetic relationship between the subfamilies recovered in this study: Agapanthoideae sister to an Allioideae-Amaryllidoideae clade is congruent with previously published studies based on plastid DNA data (Fay et al., 2000; Givnish et al., 2005; Givnish et al., 2018; Pires et al., 2006; Seberg et al., 2012). Moreover, while our sampling represents only a small fraction of the diversity within Amaryllidoideae, the relationship within the subfamily is also broadly congruent with previous studies (Meerow et al., 2006); Rønsted et al., 2012). The long branch leading to *Allium* in our phylogenetic tree is also present in studies using fewer (one to four) plastid genes (Givnish et al., 2005; Seberg et al., 2012; Chen et al., 2013); the reason behind this is beyond the scope of our paper.

## Conclusions

We are still very much in the discovery stage when it comes to patterns of gene loss and duplication in the plastome. Genes linked to a range of functions are implicated in plastome change however, not surprisingly, most of the genes that vary are linked to photosynthesis at one level or another. The loss of so many genes is *Strumaria truncata* is notable and worthy of further phylogenetic investigation through deeper taxon sampling. At this stage we are not in a position to propose driving mechanisms for the change, or whether those observed are to some extent evolutionarily neutral to the growing conditions the plants experience. While the family is characterised by bulbous geophytes, the habitats those plants occupy vary enormously from arid through mesic to seasonally inundated. Likewise, the light levels tolerated by different species vary from intense sun to quite deep shade. This paper offers a first look at the kinds of variation in plastomes that might be found across the family and indicates this will be a promising area for more detailed investigation.

## Supplemental Information

10.7717/peerj.12400/supp-1 Supplemental Information 1 Alignment of *Strumaria truncata* and *Nerine sarniensis* plastomes in Geneious Prime 2020.0.5, showing: (A)the extracted regions between *trnN* and *trnN* in the IRs, including the complete SSC; (B) J_SB_ and (C) J[sub] Red boxes highlight the alignment of the 45 bp sequence region downstream of the *ndhH* pseudogene present in the *S. truncata* IRs. Green bars under ‘Consensus’ indicate 100% pairwise sequence identity, greeny-brown bars > 30% and red bars < 30% pairwise sequence identity. Lack of any bars indicate SNPs and indels in panels B and C. Green arrows indicate gene annotations, yellow arrows show protein coding reading frames, purple arrows show tRNAs, and orange arrows are IR annotations.

10.7717/peerj.12400/supp-2 Supplemental Information 2 Details of the PCR primers used to amplify and sequence junctions of the inverted repeats and the *ndh* gene sequences in the *Strumaria truncata* plastome assembly Primers for this study were designed with the Primer3 plugin in Geneious v11.1.5. PCR primers are highlighted in bold, all other primers used as internal sequencing primers.

10.7717/peerj.12400/supp-3 Supplemental Information 3 Details of PCR cycling conditions for the amplification of the inverted repeat junctions and the *ndh* gene sequences in the *Strumaria truncata* plastome assembly

10.7717/peerj.12400/supp-4 Supplemental Information 4 Details of reading frame amendments for the Amaryllidaceae plastomes from GenBank

10.7717/peerj.12400/supp-5 Supplemental Information 5 Assembly details for the different plastome assembly strategies. ’/’ indicates that the two NOVOPlasty outputs differed in length

## Additional Information and Declarations

### DNA Deposition

The following information was supplied regarding the deposition of DNA sequences: Raw sequence data are available in SRA (BioProject: PRJNA730513); assembled plastomes are available on GenBank (MN539611–MN539615).

### Data Availability

The following information was supplied regarding data availability: Sequence alignment of the 67 protein coding genes used in the phylogenetic analysis and the resulting newick tree is available at GitHub: https://github.com/kalmankonyves/Amaryllidaceae_plastomes-Strumaria.
