# Supplementary material for: Comparative plastomics of Amaryllidaceae: inverted repeat expansion and the degradation of the ndh genes in Strumaria truncata Jacq
Source: PeerJ. 2021 Nov 12;9:e12400. doi: 10.7717/peerj.12400 (PMC8592052; doi:10.7717/peerj.12400)
Supplement: Supplemental Information 2 — Primers for this study were designed with the Primer3 plugin in Geneious v11.1.5. PCR primers are highlighted in bold, all other primers used as internal sequencing primers. [file peerj-09-12400-s002.docx]

**Table S1.** Details of the PCR primers used to amplify and sequence junctions of the inverted repeats and the *ndh* gene sequences in the *Strumaria truncata* plastome assembly. Primers for this study were designed with the Primer3 plugin in Geneious v11.1.5. PCR primers are highlighted in bold, all other primers used as internal sequencing primers.

| **Application** | **Primer** | **Sequence** | **Source** |
| --- | --- | --- | --- |
| Strumaria SSC/IRa | **ndhI_470F** | **CTTTGACCAATGTACCTTGC** | This study |
|  | ndhI_rps15_855F | AAATTCATGATAAGAATTCC | This study |
|  | ndhI_rps15_1250R | TTAGGAACATCAAGGTACAC | This study |
|  | ndhI_rps15_1387F | CTTAATCTTGGAGTAAACAG | This study |
|  | ndhI_rps15_1500R | TGATGAGTTAAGCTAGATAG | This study |
|  | ndhI_rps15_1723F | ATTATTCGATCCATATCCTG | This study |
|  | ndhI_rps15_2334F | TCAGTCTAAGAACACCATGC | This study |
| Strumaria SSC/IRb | **rpl32_120F** | **GGTAGAAACTGACTTTGCTAA** | This study |
|  | rps15_rpl32 | AATTGAATAAGTTCATAGTG | This study |
| Strumaria SSC/IRa & IRb | ndhI_rps15_1983R | ATTGTTATATGATCTATTCG | This study |
|  | rps15_95R | CRGAGACTYACTTCACATTTGG | This study |
|  | **ycf1_end** | **TTTTGAACGAGGAAAAGCAT** | This study |
| Strumaria ndh1 | **ccsA_F** | **ACAAATCAAAGTTTGCAAGGTA** | This study |
|  | **psaC_R** | **TAGGATGTACTCAATGTGTACG** | This study |
|  | ndhD_F | ATCCATAGAACATCTGACGT | This study |
|  | ndhD_R | TTACTGACAGGATTTATCAC | This study |
| Strumaria ndh2 | **ndhJ_F** | **ATGAAATGACAGCAATGGAGTC** | This study |
|  | **ndhC_R** | **CCGAATCCGCTATTACATGTTT** | This study |
| Strumaria ndh3 | **psaC_2F** | **CAATAAGATAGACCCATGCTGC** | This study |
|  | **rps15_ndhI_R** | **ACTTCTTCCCAACTTGTTTCAC** | This study |
|  | ndhI_R | GAATTTGATAAATGTATTGC | This study |
|  | psaC_ndhI_F | GTTAGTATTTGTAGTTTCCG | This study |
| LSC/IRa | **rpl2_F** | **CAGAGGGGCTATAATTGGAGAT** | This study |
|  | **psbA_R2** | **CGTCCTTGGATTGCTGTTG** | This study |
|  | psbA_R1 | CCTTGGTATGGAAGTAATGC | This study |
|  | rpl2-psbA-F3 | GGTAARCGYCCYGTAGTAAGAGG | Wang et al. [(2008)](https://paperpile.com/c/vpeL2L/4pPy/?noauthor=1) |
| LSC/IRb | **rps3-F1** | **ATAWATTCYGCAAGAATRTTAGG** | Wang et al. (2008) |
|  | **rps3-rpl2-R1** | **AATGGGAAATGCCCTACCTTTG** | Wang et al. (2008) |
|  | rps3-F2 | AGTCKGAAACCRAGTGGATTT | Wang et al. (2008) |
|  | rps3-rpl2-R2 | GTAGTAAGAGGRGTRGTTATGAACCC | Wang et al. (2008) |
| LSC/IRb for Nerine and Strumaria | **rps3-F1_G** | **ATAWATTCYGCAAGAATRTTGGG** | Modified from  Wang et al. (2008) |
| SSC/IRa & IRb | **trnN** | **CTCTACCACTGAGCTACTGAG** | This study |
|  | cp211R_Mod | ACCAAGTTCAACGTTAGCCAGA | Modified from  Stoll et al. [(2017)](https://paperpile.com/c/vpeL2L/N1Uz/?noauthor=1) |
| SSC/IRa | **cp262_Mod** | **TTTGTATGACCAYCGAGAAACC** | Modified from  Stoll et al. (2017) |
|  | ycf1_F | GCGATTCCATCGTTTATAATCG | This study |
| SSC/IRb | **ndhF_1318F** | **GGATTAACCGCATTTTATATGTTTC** | Terry et al. [(1997)](https://paperpile.com/c/vpeL2L/eS6E/?noauthor=1) |
|  | ndhF_end | GGTCATATAATCGTGGTTAC | This study |
| SSC/IRa poeticus type | **ycf1_888** | **ATACGACATTGATTGACTCTAT** | This study |
|  | ycf1_211 | TGGTCGATTCGTGGATAATC | This study |
| SSC/IRb poeticus type | **Pycf1_872** | **GCGTGGTAATTTATACTGATAA** | This study |
|  | Pycf1_192 | AGATTCTATTTCCTTTCTGTCTGA | This study |

[Stoll A, Harpke D, Schütte C, Stefanczyk N, Brandt R, Blattner FR, Quandt D. 2017. Development of microsatellite markers and assembly of the plastid genome in Cistanthe longiscapa (Montiaceae) based on low-coverage whole genome sequencing. *PloS One* 12:e0178402. DOI:](http://paperpile.com/b/vpeL2L/N1Uz) [10.1371/journal.pone.0178402.](http://dx.doi.org/10.1371/journal.pone.0178402.)

[Terry R, Brown G, Olmstead R. 1997. Examination of subfamilial phylogeny in Bromeliaceae using comparative sequencing of the plastid locus ndhF. *American Journal of Botany* 84:664.](http://paperpile.com/b/vpeL2L/eS6E)

[Wang R-J, Cheng C-L, Chang C-C, Wu C-L, Su T-M, Chaw S-M. 2008. Dynamics and evolution of the inverted repeat-large single copy junctions in the chloroplast genomes of monocots. *BMC Evolutionary Biology* 8:36. DOI:](http://paperpile.com/b/vpeL2L/4pPy) [10.1186/1471-2148-8-36.](http://dx.doi.org/10.1186/1471-2148-8-36.)
