# Supplementary material for: Comparative plastomics of Amaryllidaceae: inverted repeat expansion and the degradation of the ndh genes in Strumaria truncata Jacq
Source: PeerJ. 2021 Nov 12;9:e12400. doi: 10.7717/peerj.12400 (PMC8592052; doi:10.7717/peerj.12400)
Supplement: Supplemental Information 3 [file peerj-09-12400-s003.docx]

**Table S2.** Details of PCR cycling conditions for the amplification of the inverted repeat junctions and the *ndh* gene sequences in the *Strumaria truncata* plastome assembly.

|  | **Initial denaturation (temp/time)** | **Denaturation (temp/time)** | **Annealing (temp/time)** | **Extension (temp/time)** | **Final extension (temp/time)** | **No. of cycles** |
| --- | --- | --- | --- | --- | --- | --- |
| Strumaria SSC/IR | 94°C/120s | 94°C/60s | 52°C/30s | 72°C/240s | 72°C/7mins | 35 |
| Strumaria ndh1 |  | 94°C/60s | 53°C/30s | 72°C/180s |  | 30 |
| Strumaria ndh2 |  | 94°C/30s | 55°C/30s | 72°C/90s |  | 30 |
| Strumaria ndh3 |  |  |  |  |  |  |
| LSC/IRa |  | 94°C/60s | 56°C/30s | 72°C/180s |  | 30 |
| LSC/IRb |  | 94°C/60s | 48°C/30s | 72°C/180s |  | 35 |
| SSC/IR |  | 94°C/60s | 60°C/30s | 72°C/180s |  | 30 |
| SSC/IR poeticus type |  | 94°C/60s | 52°C/30s | 72°C/180s |  | 30 |
