# Supplementary material for: Comparative plastomics of Amaryllidaceae: inverted repeat expansion and the degradation of the ndh genes in Strumaria truncata Jacq
Source: PeerJ. 2021 Nov 12;9:e12400. doi: 10.7717/peerj.12400 (PMC8592052; doi:10.7717/peerj.12400)
Supplement: Supplemental Information 4 [file peerj-09-12400-s004.docx]

**Table S3.** Details of reading frame amendments for the Amaryllidaceae plastomes from GenBank.

| **sample** | **gene** | **original coordinates** | **new coordinates** | **note** |
| --- | --- | --- | --- | --- |
| MT133568 | *atpI* | 15,131-15,382 | 15,131-15,874 |  |
| MN857162 | *rpoC1* | 21,194-22,492 | 21,194-22,801; 23,558-23,989 | added missing exon |
| MN857162 | *petB* | 77,128-77,133; 78,038-78,679 | 77,245-77,250; 78,038-78,679 |  |
| MN158120 | *atpI* | 15,235-15,678 | 15,235-15,978 |  |
| MN158120 | *ycf1* | 30,885-30,989 | N/A | renamed to *psbM* |
| MH118290 | *atpI* | 15,200-15,451 | 15,200-15,943 |  |
| MH118290 | *psbM* | 30,857-30,982 | 30,853-30,957 |  |
| MH159130 | *rps12* | 67,887-68,000; 137,996-138,238 | 67,887-68,000; 137,996-138,227; 138,770-138,795 | added missing exon |
|  |  | 67,887-68,000; 97,088-97,330 | 67,887-68,000;  96,531-96,556; 97,099-97,330 |  |
| MH159130 | *petD* | 75,432-75,995 | 74,728-74,735; 75,482-75,995 | added missing exon |
| MH159130 | *rpl16* | 79,500-79,910 | 79,500-79,910; 80,957-80,965 | added missing exon |
