# Supplementary material for: Comparative plastomics of Amaryllidaceae: inverted repeat expansion and the degradation of the ndh genes in Strumaria truncata Jacq
Source: PeerJ. 2021 Nov 12;9:e12400. doi: 10.7717/peerj.12400 (PMC8592052; doi:10.7717/peerj.12400)
Supplement: Supplemental Information 5 [file peerj-09-12400-s005.docx]

**Table S4.** Assembly details for the different plastome assembly strategies. '/' indicates that the two NOVOPlasty outputs differed in length.

|  | | | | **NOVOPlasty 2.7.0** | **Fast-Plast 1.2.6** | | | |
| --- | --- | --- | --- | --- | --- | --- | --- | --- |
|  |  |  |  |  | **5M** | **10M** | **20M** | **all** |
| **Species** | **Total PE reads** | **Final assembled length (bp)** | **Coverage of final assembly** | **length (bp)** | **length (bp)** | **length (bp)** | **length (bp)** | **length (bp)** |
| *Acis autumnalis* var*. oporantha* | 19,015,437 | 157,839 | 786 x | 157,881/157,839 | 159,003 | 159,021 | 157,839 | 159,035 |
| *Lapiedra martinezii* | 19,971,468 | 159,022 | 708 x | 159,022 | 146,095 | 160,059 | 160,021 | 160,059 |
| *Nerine sarniensis* | 21,031,067 | 158,312 | 1,379 x | 158,312 | 159,609 | 160,626 | 160,526 | 159,540 |
| *Pancratium maritimum* | 24,547,050 | 160,123 | 936 x | 160,123 | 160,123 | 160,123 | 160,123 | 160,123 |
| *Strumaria truncata* | 23,946,383 | 157,566 | 1,197 x | 157,566 | Longest contig: 180,959 | Longest contig: 181,201 | Longest contig: 181,186 | Longest contig:  181,086 |
